# Emotional Intelligence in Physical Activity, Sports and Judo: A Global Approach

**DOI:** 10.3390/ijerph18168695

**Published:** 2021-08-17

**Authors:** Jorge Acebes-Sánchez, Cecilia Blanco-García, Ignacio Diez-Vega, Daniel Mon-López, Gabriel Rodriguez-Romo

**Affiliations:** 1 Faculty of Health Sciences, Universidad Francisco de Vitoria (UFV), Pozuelo de Alarcón, 28223 Madrid, Spain; 2 Faculty of Physical Activity and Sport Sciences (INEF), Universidad Politécnica de Madrid, 28040 Madrid, Spain; cecilia.blanco@upm.es (C.B.-G.); daniel.mon@upm.es (D.M.-L.); gabriel.rodriguez@upm.es (G.R.-R.); 3 Departamento de Enfermería y Fisioterapia, Facultad de Ciencias de la Salud, Universidad de León, 24007 León, Spain; ignacio.diez@unileon.es; 4 CIBERFES, 28029 Madrid, Spain

**Keywords:** TMMS-24, emotional attention, emotional clarity, emotional repair, exercise, GPAQ

## Abstract

Emotional intelligence (EI) has been studied in relation to health and physical activity (PA) or in a sport-specific approach. EI is related to sports performance; however, combat sports seem to show characteristics of their own that involve better control of emotions than other sports. This study aimed to analyse whether there are differences in EI dimensions between those who do not achieve World Health Organization (WHO) PA recommendations, those who meet WHO PA recommendations, those who meet WHO PA recommendations practising sports, and judokas of different levels. A descriptive and cross-sectional study was conducted. The sample comprised 2938 undergraduate students from Madrid and 487 active Spanish judokas. PA was measured by the Global Physical Activity Questionnaire (GPAQ). EI was assessed by the Trait Meta-Mood Scale (TMMS-24). Three different one-way ANOVA and ANCOVA (controlling for gender and age) were conducted to compare the effect of type of group studied on the EI dimensions. Significant differences in EI dimensions were found between those who do not meet PA recommendations, those who meet PA recommendations, those who meet PA recommendations practising sports, and judo athletes of different levels. However, when controlling for gender and age, these differences remained specifically in emotional attention and in emotional repair. Judo athletes and high-performance judo athletes showed better EI than the rest of the studied groups.

## 1. Introduction

Emotional intelligence (EI) is the ability to perceive accurately, appraise, and express emotions; to generate feelings when they facilitate thought; to understand emotion and emotional knowledge; and to regulate emotions to promote emotional and intellectual growth [1]. There is a scientific debate on what is the most appropriate tool to assess EI [2]. Petrides and Furnham [3] differentiated between trait EI and information-processing EI. The “information-processing EI” refers to the ability to identify, express, and label emotions. This understanding of EI is evaluated through measures of peak (not typical) performance, such as the Emotional Intelligence Test (MSCEIT) by Mayer et al. (2003). Trait EI is related to the consistency in a specific behaviour characterized by empathy, assertiveness, or optimism and would therefore be embedded in personality. This type of EI is assessed through validated self-report inventories (e.g., Bar-On Emotional Quotient Inventory (EQ-i), Bar-On, 1997 [4]; Trait Emotional Intelligence Questionnaire (TEIQue) [5]). This study is interested in the stable affective capacities that athletes routinely use to experience their feelings and moods [6,7]. This approach is denominated as Trait Meta Mood divided into three emotional dimensions (attention, clarity, and repair). To measure it, Salovey et al. [6] created the Trait Meta Mood Scale (TMMS-24), becoming one of the most widely used tools because of its ease of use [8,9]. The TMMS-24 has been validated in Spanish [10] and is one of the few tools validated in a sports context [11].

According to previous studies, EI is positively related to general health, mental health, and physical health [12]. EI also correlates positively with positive mood [13] and lower stress [14]. Different studies showed that women have higher levels of EI compared with men [15]. However, some studies have found that women had significantly greater emotional attention and that men had greater emotional clarity and repair [16]. On the other hand, it seems that EI increases progressively with age [17].

In recent years, EI has been studied in the physical activity (PA) and sport context. A systematic review by Laborde et al. [18] showed that the vast majority of the studies reviewed focused on the possible links between EI and sport, with fewer studies looking at the links between EI and PA. Instead, the systematic review also showed that 34 of the 36 reviewed studies used self-report tools to assess EI (trait or mixed model). Studies analysing the relationship between EI and PA levels are limited, and methodological differences have been found between them, making it difficult to compare their results [18]. In most cases, the reviewed studies showed a positive association between EI and PA levels. Thus, those who achieved PA recommendations showed higher EI than those who do not [9,19,20]. Other research has shown significant positive correlations between EI and the amount of exercise performed [21,22,23,24,25,26]. At the sociodemographic level, some research shows that women have higher EI scores than men [15]. However, according to PA levels, there are differences in EI between men and women who practise PA and those who do not, which is significantly higher in those who practise PA [16], so sociodemographic variables should be controlled for in the study of these associations. On the other hand, a systematic review by Ubago-Jiménez et al. [27] of EI and PA pointed out that the majority of the studies are in an educational environment.

Concerning EI and sports relationships, Zamian et al. [28] point out that the practice of sport brings emotions into play since they must constantly control and manage their emotions under different conditions of training and competition. Campo et al. [29] suggested that the practice of sports could be a mechanism for the development of EI. Thus, each athlete experiences a sporting experience differently on an emotional level. Several studies have revealed a positive correlation between EI and sports performance [30,31,32]. Kopp and Crombie [33,34] even proposed EI as a predictor of sports performance. Some researchers have proposed comparative studies between athletes and non-athletes, finding that athletes have significantly higher EI than non-athletes [35,36]. The same differences have specifically also found in female athletes and non-athletes [37,38].

Different research on EI has been developed specifically in the context of sports. Laborde et al. [18] pointed out that different psychological requirements are needed according to the type of sport. Diverse studies showed no significant differences in EI according to the type of sport [20,39]. Nevertheless, Castro-Sánchez et al. [40] found higher EI levels in team sports than in individual ones. In most sports disciplines, maximum effort is required from athletes during competition, situations of mental stress and physical fatigue [41]. However, in the case of combat sports, other circumstances of a more specific nature must also be added. Psychological and personality traits of the athletes as well as their cognitive abilities are particularly important aspects of performance. Some of these characteristics are the physical contact and direct attack on the opponent’s body, the usual pre-competition weight loss, the risk of experiencing pain and possible injuries, as well as the persistent fear of failure in the face of the opponent. Martial arts have a specific philosophy, a hierarchical system, and dojo rules that provide a particularly differentiating environment to develop personal values and skills [42,43], such as EI. Among the benefits of practising martial arts, some authors mention the ability to control emotions and the ability to self-control reaction in case of danger [44,45]. Ghoul [46] argued that low EI affects numerous dimensions of sports performance in combat sports. Piskorska et al. [41] exposed that emotions are essential in this context since that they determine arousal during competition. It is especially important in combat sports where fighting an opponent may be accompanied by a risk of pain or injury. Emotions such as fear may paralyze the athlete and reduce their ability to make the best decisions [47]. Different studies conclude that combat athletes have better EI than non-athletes [35,48]. On the other hand, two studies conducted with combat athletes of different modalities showed that those athletes who competed at a higher level showed better levels of EI [8,49]. Some authors believe that this may be because combat sports training may have a positive impact on anxiety control and the development of EI [41]. Nakamura, Kodama and Mukaino [50] developed research aimed at understanding the mental characteristics of combat athletes. The participants were boxing, kick-boxing, judo, and wrestling athletes. These authors concluded, on the one hand, that a mental characteristic of combat sports practitioners is the tendency to be susceptible to anxiety and, on the other hand, that the specific characteristics of each of the sports examined leads to differences in different areas of emotional intelligence.

In the literature, studies have compared individuals who achieved PA recommendations vs. those who do not [9,19], those who practise sport vs. those who do not [35,36], or EI levels according to the type of sport [20,51]. In many cases, it was concluded that both PA and sport are appropriate mediums for the work and development of EI. However, these have always been partial studies and, to the best of our knowledge, no study compares all of these subgroups together and offers a more comprehensive view of possible differences between them in terms of IE. The fact is that assessing these groups together allows us to evaluate the extent to which PA; sports in general; and sports in particular, such as Judo, contribute to the development of EI. Therefore, this study aimed to analyse whether there are differences in EI dimensions between those who do not meet PA recommendations, those who meet PA recommendations, those who meet PA recommendations specifically practising sports, and judokas of different levels.

## 2. Materials and Methods

### 2.1. Design and Participants

A descriptive and cross-sectional study was conducted using an online questionnaire designed for the investigation as a data collection instrument. The sample comprised undergraduate students from public and private universities in Madrid, excluding online bachelor’s degree students. Among them, 1772 undergraduate students claimed to achieve WHO recommendations practising sports. All those who reported practising combat sports were eliminated from the sample. The sample also comprised active judo Spanish athletes with three years minimum experience in judo, of which 129 were considered high-performance athletes. These athletes, according to Spanish law had a valid certificate of “Deportista de Alto Nivel” (high-level athlete) and/or “Deportista de Alto Rendimiento” (high-performance athlete). This certificate is conditional on high sporting performance. Participation was voluntary and confidential, and informed consent was obtained from participants before they completed the survey.

### 2.2. Data Collection

Different lecturers from 6 public and 7 private universities were contacted by email, the contact details were public on the internet. Some lecturers decided to participate voluntarily in the study by sending to their students a Google Forms Questionnaire. On the other hand, judo athletes were contacted through their regional federations; in addition, the heads of the most important clubs at the national level were contacted. The questionnaire was available online from October 2020 to December 2020. Once the questionnaire was closed, the answers were checked. Contradictory responses and empty questionnaires were deleted. Sociodemographic (gender and age) information was collected to examine the possible moderating effect of these variables.

### 2.3. Measurement of Variables

#### 2.3.1. Global Physical Activity Questionnaire (GPAQv2)

PA was measured by the Global Physical Activity Questionnaire version 2 (GPAQv2), which contains 16 questions and captures information about PA in a typical week [52]. This questionnaire provides information about the intensity (moderate or intense), frequency (days in a typical week), and duration (hours and minutes in a typical day) of physical activity performed across its three domains: (i) occupational: paid or unpaid job, studies, housework, or job search (OPA); (ii) commute-related: walking or cycling (CPA); and (iii) leisure-time (LTPA). It also contains a question assessing the time (in minutes) spent sitting or reclining during a typical day.

The GPAQ derives from the International Physical Activity Questionnaire (IPAQ), which has been validated and widely used to assess PA patterns [53]. The GPAQ shows good reliability; positive moderate to strong correlation with the IPAQ; and a validity, albeit low, similar to that of other subjective tools designed to evaluate PA patterns including the IPAQ itself [54].

Here, we used the Spanish version of the GPAQv2 without modifying the original contents or text of the questionnaire. The GPAQ protocol was strictly adhered to for data recompilation and treatment [52].

From the duration (minutes), intensity (moderate, vigorous), and frequency (days per week) of physical activities performed in a typical week, PA-related energy expenditure was calculated according to the guidelines of the questionnaire’s data treatment protocol [52]. Total PA (sum of OPA, CPA, and LTPA) was classified into three levels (high, moderate, and low) according to the time spent on PA per day in a typical week, the number of days on which this PA was performed, and the intensity of this PA [52] (see Table 1). According to the World Health Organization (WHO), adults aged 18–64 years should perform at least 150 min of moderate-intensity aerobic physical activity or at least 75 min of vigorous-intensity aerobic physical activity per week or an equivalent combination of moderate- and vigorous-intensity activity. The cut-offs used to establish these three groups were based on PA recommendations [55]. Thus, the participants assigned to the low PA level were defined as insufficiently active, i.e., they performed no PA or did not fulfil the minimum recommendations for PA to obtain a health benefit. In contrast, those assigned to the moderate and high PA levels were individuals who were sufficiently active, i.e., those who fulfilled or exceeded these recommendations.

To calculate the levels (high, moderate, and low) of PA in each area or domain (occupational, commuting, and leisure time), the same criteria were followed as those outlined for total PA, although only PA carried out in the area in question was considered.

#### 2.3.2. Trait Meta Mood Scale 24 (TMMS-24)

The sample was assessed by the Spanish version of the Trait Meta-Mood Scale (TMMS-24), based on the original scale developed by Salovey et al. [6]. Its self-report assessment contained 24 items with a five-point Likert scale from 1 (totally disagree) to 5 (totally agree). It is composed of three dimensions (8 items each dimension): (i) emotional attention (EA), evaluating how people attend to and value their feelings (item example: “I pay a lot of attention to my feelings”); (ii) emotional clarity (EC), which is how people feel clear rather than confused about their feelings (item example: “I can often define my feelings”; (iii); and emotional repair (ER), which is how people use positive thinking to repair negative moods (item example: “Although sometimes I feel sad, I usually have an optimistic vision”. For EA, scores between 22–32 for men and 25–35 for women are adequate EA. Higher or lower scores mean worse EA, either too little or too much. Men with scores below 26 and women with scores below 24 should improve their EC. Scores over 26 in men and over 24 in women have adequate EC. Men and women with scores below 24 should improve their ER. Scores over 24 for men and women mean adequate ER. Scores over 36 in men and over 35 in women mean excellent EC or ER. This Spanish version was validated by Fernández-Berrocal et al. [10]. Our results demonstrate similar internal consistency in the three sub-scales: EA, α = 0.89; EC, α = 0.9; and ER, α = 0.83.

### 2.4. Statistical Methods

The data collected by the questionnaires were analysed using the Statistical Package for the Social Sciences (SPSS v21) (IBM, Armonk, NY, USA). A descriptive analysis was performed to explore the sample characteristics.

The sample characteristics are described by frequency, percentages, mean (M), and standard deviation (SD). The sample was divided into 5 groups: Group 1: Does not achieve WHO-Recommended level of PA; Group 2: Achieved WHO-Recommended level of PA (do not practise sport); Group 3: Achieved WHO-Recommended level of PA (practise sport); Group 4: Judo athletes; and Group 5: High-performance judo athletes. Three different one-way ANOVAs were conducted to compare the effect of type of group studied on the EI dimensions (EA, EC, and ER). Three different one-way ANCOVAs were conducted to determine a statistically significant difference between the type of group studied on EI dimensions (EA, EC, and ER) controlling for gender and age. Statistical significance was set at *p* < 0.05. However, as EI consists of three dimensions, the significance level was adjusted at *p* < 0.017. A post hoc analysis was conducted using the Bonferroni test. We used η^2^_p_ to interpret the effect size, defining values under 0.06 as a small effect, values between 0.06 and 0.14 as a moderate effect, and values above 0.14 as a large effect [56].

## 3. Results

### 3.1. Characteristics of the Sample

Participation was voluntary following a free e-mail campaign. The sample was composed of 2938 undergraduate students (21.13 ± 3.47 years: 21.16 ± 3.42 years for men and 21.12 ± 3.50 years for women). Another group of 487 judo athletes was included (30.22 ± 9.50 years: 31.27 ± 9.71 years for men and 28.76 ± 8.95 years for women). Table 2 shows the characteristics and distribution of the sample.

### 3.2. Emotional Attention

The analysis of variance shows a small significant effect of belonging to one of the study groups on EA, *F*(4,3420) = 7.23, *p* ≤ 0.001, η_p_^2^ = 0.008. Post hoc comparisons using the Bonferroni test indicated that the EA mean score for the group that does not achieve the WHO-Recommended level of PA (*M* = 29.41; *SD* = 6.31) was significantly different from the group who achieved the WHO-Recommended level of PA and practises sports (*M* = 28.70; *SD* = 6.15), judo athletes (*M* = 28.89; *SD* = 6.00), and high-performance judo athletes (*M* = 27.14; *SD* = 6.53). The EA mean score for the group that achieves the WHO-Recommended level of PA but does not practise sports (*M* = 28.91; *SD* = 6.17) was significantly different from high-performance judo athletes (*M* = 27.14; *SD* = 6.53). The EA mean score for the group that achieves the WHO-Recommended level of PA and practises sports (*M* = 28.70; *SD* = 6.15) was significantly different from high-performance judo athletes (*M* = 27.14; *SD* = 6.53).

The one-way ANCOVA shows a small significant effect of belonging to one of the study groups on EA after controlling for gender and age, *F*(4,3420) = 3.59, *p* = 0.006, η_p_^2^ = 0.004. Gender shows a small significant effect (*p* ≤ 0.001; η_p_^2^ = 0.021). Post hoc comparisons using the Bonferroni test indicated that the EA mean score for the group that does not achieve the WHO-Recommended level of PA (*M* = 29.41; *SD* = 6.31) was significantly different from high-performance judo athletes (*M* = 27.14; *SD* = 6.53). The EA mean score for the group that achieved the WHO-Recommended level of PA but does not practise sports (*M* = 28.91; *SD* = 6.17) was significantly different from high-performance judo athletes (*M* = 27.14; *SD* = 6.53).

### 3.3. Emotional Clarity

An analysis of variance shows a small significant effect of belonging to one of the study groups on EC, *F*(4,3420) = 24.99, *p* ≤ 0.001, η_p_^2^ = 0.028. Post hoc comparisons using the Bonferroni test indicated that the EC mean score for the group that does not achieve the WHO-Recommended level of PA (*M* = 27.68; *SD* = 6.37) was significantly different from judo athletes (*M* = 28.44; *SD* = 6.11) and high performance judo athletes (*M* = 29.09; *SD* = 5.95). The EC mean score for the group that achieved the WHO-Recommended level of PA but does not practise sports (*M* = 27.46; *SD* = 6.09) was significantly different from judo athletes (*M* = 28.44; *SD* = 6.11), and high-performance judo athletes (*M* = 29.09; *SD* = 5.95). The EC mean score for the group that achieved the WHO-Recommended level of PA and practises sports (*M* = 28.04; *SD* = 6.04) was significantly different from judo athletes (*M* = 28.44; *SD* = 6.11) and high-performance judo athletes (*M* = 29.09; *SD* = 5.95).

The one-way ANCOVA does not show a significant effect of belonging to one of the study groups on EC after controlling for gender and age, *F*(4,3420) = 2.70, *p* = 0.029, η_p_^2^ = 0.003.

### 3.4. Emotional Repair

An analysis of variance shows a small significant effect of belonging to one of the study groups on ER, *F*(4,3420) = 48.91, *p* ≤ 0.001, η_p_^2^ = 0.054. Post hoc comparisons using the Bonferroni test indicated that the ER mean score for the group that does not achieve the WHO-Recommended level of PA (*M* = 27.17; *SD* = 6.25) was significantly different from the group that achieves the WHO-Recommended level of PA and practises sports (*M* = 28.66; *SD* = 5.57), judo athletes (*M* = 29.00; *SD* = 5.71), and high performance judo athletes (*M* = 30.75; *SD* = 5.05). The ER mean score for the group that achieves the WHO-Recommended level of PA but does not practise sports (*M* = 27.16; *SD* = 6.14) was significantly different from the group that achieved the WHO-Recommended level of PA and practises sports (*M* = 28.66; *SD* = 5.57), judo athletes (*M* = 29.00; *SD* = 5.71), and high-performance judo athletes (*M* = 30.75; *SD* = 5.05). The ER mean score for the group that achieves the WHO-Recommended level of PA and practises sports (*M* = 28.66; *SD* = 5.57) was significantly different from judo athletes (*M* = 29.00; *SD* = 5.71), and high-performance judo athletes (*M* = 30.75; *SD* = 5.05).

The one-way ANCOVA shows a small significant effect of belonging to one of the study groups on ER after controlling for gender and age, *F*(4,3420) = 18.40, *p* ≤ 0.001, η_p_^2^ = 0.021. Gender shows a small significant effect (*p* ≤ 0.001; η_p_^2^ = 0.005). Age shows a small significant effect (*p* ≤ 0.001; η_p_^2^ = 0.023). Post hoc comparisons using the Bonferroni test indicated that the ER mean score for the group that do not achieve the WHO-Recommended level of PA (*M* = 27.17; *SD* = 6.25) was significantly different from the group that achieved the WHO-Recommended level of PA and practises sports (*M* = 28.66; *SD* = 5.57), judo athletes (*M* = 29.00; *SD* = 5.71), and high-performance judo athletes (*M* = 30.75; *SD* = 5.05). The ER mean score for the group that achieved the WHO-Recommended level of PA but does not practise sports (*M* = 27.16; *SD* = 6.14) was significantly different from the group who achieved the WHO-Recommended level of PA and practises sports (*M* = 28.66; *SD* = 5.57), judo athletes (M = 29.00; SD = 5.71), and high-performance judo athletes (*M* = 30.75; *SD* = 5.05). The ER mean score for the group that achieved the WHO-Recommended level of PA and practises sports (*M* = 28.66; *SD* = 5.57) was significantly different from high-performance judo athletes (*M* = 30.75; *SD* = 5.05).

Table 3 shows the differences among the groups studied in EA dimensions (EA, EC, and ER) using one-way ANOVA for crude analysis and ANCOVA controlling for gender and age.

## 4. Discussion

This study analysed the differences in EI dimensions (EA, EC, and ER) between those who do not meet PA recommendations, those who meet PA recommendations, those who meet PA recommendations specifically practising sports, and judo athletes of different levels.

It appears that our research has found differences in ER between all groups. Differences controlling for gender and age remain. Thus, those who do not meet the recommendations have significantly less ER than those who meet PA recommendations by doing sport, judo athletes, and high-performance judo athletes. Those who achieved the PA recommendations without practising sports have significantly less ER than those who meet the recommendations by doing sport, judo athletes, and high-performance judo athletes. Additionally, those who meet the PA recommendations by doing sport have significantly less ER than high-performance judokas. In other words, those who practise sport have significantly higher ER. Judokas have significantly higher ER levels compare with those who meet PA recommendations and practice sports. If judo is practised at the high-performance level, ER is even higher. Different studies showed similar results, where high-level athletes showed significantly higher ER results than low-level athletes. These results were in combat sports [8,49] and canoeists [57]. It is maybe because high-level athletes have more experience and are in a competitive atmosphere. To reach the competitive level, they have had to win and lose on numerous occasions, overcoming problems of many kinds. Salovey et al. [6] established that ER is associated with control intrusive and ruminative thoughts that often accompany stressful situations. In the sports context, athletes who recover from negative emotional will compete better. Different techniques have been developed by sports psychologists aimed at achieving optimal performance [58]. These strategies to regulate emotions become crucial in the sports context [59]. Instead, our findings also support the results obtained by other studies that compared athletes with non-athletes and found significantly superior stress management in athletes [60]. Pasand et al. [61] showed that athletes in their study scored significantly higher on problem-solving. All of these variables are related to ER. Furthermore, the differences between judo athletes and practitioners of other sports support the idea by Reche-García et al. [62], who concluded that practitioners of combat sports had significantly higher levels of resilience than individual or team sports athletes. As mentioned above, combat sports have some differentiating characteristics from other sports that can be related to working on and improving ER [41].

Our results showed that those who do not meet PA recommendations have significantly higher EA than those who meet PA recommendations practising sports, judo athletes, and high-performance judo athletes. However, controlling by gender, these significant differences are only present with the group of high-performance judo athletes. These results suggest that individuals who do not meet PA recommendations may have a problem since higher EA is related to poorer emotional adjustment [63] and excessive reactions to negative emotions [64]. High EA is related to high repletion of negative thoughts. As indicated by Roy et al. [65], athletes in dynamic, team sports might benefit from the flexibility associated with being low in rumination. Some authors state that, specifically in combat sports, a greater psychological defence is developed. This contributes to avoiding anxiety and any other negative emotions, blocking the real impact of certain threats [66].

Different studies have shown that athletes show better results in EI than non-sport athletes [35,36]. However, only the research developed by Costarelli and Stamou [48] studied these differences with high-performance taekwondo and judo athletes compared with non-athletes, with similar results, being significantly higher in EI for high-performance athletes, specifically in assertiveness and emotional flexibility.

Although our results have shown that there are significantly higher EC levels for judo athletes and high-performance judokas compared with the rest of the groups, these differences disappear when controlling the gender and age of the sample. These results are not in line with studies conducted by Merino et al. [8,49], who found that high-performance combat sports athletes showed significantly higher levels of EC than lower-level athletes. It should be noted that the levels of EC in all groups according to the assessment tool are adequate [10]. However, those judo athletes and high-performance judo athletes are closer to excellent. In this sense, it does not seem that participation in sports, or judo specifically, as well as a high performance in this sport provide a context that enhances emotional clarity in any way. On the other hand, in the case of emotional clarity, it seems that gender and age are more relevant than practicing sport, specifically judo [16,17].

The present study has certain limitations that need to be acknowledged. The cross-sectional design means that we were unable to infer causal relationships among the analysed variables, specifically the analyses do not provide causality in the relationships between sports activity and EI development. Another limitation was the absence of generalization to other sports. Longitudinal studies would be required to establish cause--effect relationships and to study EI changes during sports practise and in different competitions contexts. Our research also has important strengths. On the one hand, it offers a joint vision, hitherto non-existent, of the possible relationships between EI and those who do not meet PA recommendations, those who meet PA recommendations, those who practise sports, and judokas of different levels. On the other hand, our research was carried out with a large sample of undergraduate students and judo athletes of different levels using the TMMS-24 as a measurement instrument. It should be noted that effect sizes were low in all significant associations. Lastly, we consider that our results support the idea that the practice of PA, specifically sport, leads to an improvement in psychological variables, such as EI. On the other hand, specifically in judo, due to its internal philosophy, could develop the EI of its practitioners. Likewise, those who become high-performance athletes seem to show better EI levels, which is a point to be taken into account by coaches and sports psychologists. Future studies should investigate what circumstances make combat sports a different sport for EI development. As well as studying the related variables in high-performance athletes that are related to better performance.

## 5. Conclusions

The main conclusion of this study is that there are significant differences in EI (EA, EC, and ER) between those who do not meet PA recommendations, those who meet PA recommendations, those who meet PA recommendations specifically practising sports, and judo athletes of different levels. However, when controlling the gender and age of the participants, these differences remain specifically in EA and in ER. Judo athletes and high-performance judo athletes have better EI than those who do not meet PA recommendations, those who meet PA recommendations, and those who meet PA recommendations specifically practising sports.

## Figures and Tables

**Table 1:** Total physical activity levels and classification criteria recommended for use with the Global Physical Activity Questionnaire.

Level of Total Physical Activity	Physical Activity Thresholds
High	≥3 days of vigorous activity (at work and during recreation) in a typical week AND a total physical activity MET-minutes per week ≥1500. ≥7 days of vigorous activity (during commute, at work, and during recreation) in a typical week AND a total physical activity MET-minutes per week ≥3000.
Moderate	Does not achieve the above criteria but does achieve one of the next following: (a) ≥3 days of vigorous activity (at work and during recreation) in a typical week, at least 20 min each day. (b) ≥5 days of vigorous or moderate activity (during commute, at work, and during recreation) in a typical week, at least 30 min each day. (c) ≥5 days of vigorous or moderate activities (during commute, at work, and during recreation) in a typical week, at least 600 MET-minutes per week of total physical activity.
Low	Does not achieve the high or moderate criteria.

**Table 2:** Sample distribution and characteristics.

Characteristics	Age *M* *±* *SD*	*N*	%
Gender			
Male	30.81 ± 7.62	1251	36.5
Female	23.54 ± 5.26	2174	63.5
Sample distribution			
Undergraduate students	21.13 ± 3.47	2938	85.8
Judo Athletes	30.22 ± 9.50	487	14.3
Groups of study			
Does not achieve WHO-Recommended level of PA	21.35 ± 4.51	472	13.8
Achieved WHO-Recommended level of PA (do not practise sport)	21.47 ± 4.61	694	20.3
Achieved WHO-Recommended level of PA (practise sport)	21.29 ± 4.18	1772	51.7
Judo athletes	30.44 ± 9.66	358	10.5
High-performance judo athletes	29.62 ± 8.95	129	3.8

**Table 3:** Differences among groups studied in the emotional intelligence dimensions (EA, EC, and ER).

Groups	Descriptives		Crude		Adjusted	
	*M*	*SD*	*p*	η_p_^2^	*p*	η_p_^2^
Emotional Attention			0.006 *	0.008	≤0.001 *	0.004
Group 1 ^3,4,5,5′^	29.41	6.31				
Group 2 ^5,5′^	28.91	6.17				
Group 3 ^5^	28.70	6.15				
Group 4	28.89	6.00				
Group 5	27.14	6.53				
Emotional Clarity			≤0.001 *	0.028	0.029	0.003
Group 1 ^4,5^	27.68	6.37				
Group 2 ^4,5^	27.46	6.09				
Group 3 ^4,5^	28.04	6.04				
Group 4	28.44	6.11				
Group 5	29.09	5.95				
Emotional Repair			≤0.001 *	0.054	≤0.001 *	0.021
Group 1 ^3,4,5,3′,4′,5′^	27.17	6.25				
Group 2 ^3,4,5,3′,4′,5′^	27.16	6.14				
Group 3 ^4,5,5′^	28.66	5.57				
Group 4	29.00	5.71				
Group 5	30.75	5.05				

η_p_^2^ = Partial eta squared; * *p* ≤ 0.01; The adjusted model was fitted for gender and age; Group 1: Does not achieve WHO-Recommended level of PA; Group 2: Achieved WHO-Recommended level of PA (does not practise sports); Group 3: Achieved WHO-Recommended level of PA (practises sports); Group 4: Judo athletes; Group 5: High-performance judo athletes; ^3^ = Significant differences with group 3 (Crude); ^4^ = Significant differences with group 4 (Crude); ^5^ = Significant differences with group 5 (Crude); ^3^^′^ = Significant differences with group 3 (Adjusted); ^4^^′^ = Significant differences with group 4 (Adjusted); ^5^^′^ = Significant differences with group 5 (Adjusted).

## Data Availability

The data are available for those who want to see it with justified reasons. Kindly contact the corresponding author.
